# Global Trends in Intertrochanteric Hip Fracture Research From 2001 to 2020: A Bibliometric and Visualized Study

**DOI:** 10.3389/fsurg.2021.756614

**Published:** 2021-10-28

**Authors:** Ze Zhang, Yudian Qiu, Yawen Zhang, Yi Zhu, Fengpo Sun, Junchuan Liu, Tongyi Zhang, Liangyuan Wen

**Affiliations:** ^1^Department of Orthopedics, Beijing Hospital, National Center of Gerontology, Institute of Geriatric Medicine, Chinese Academy of Medical Sciences, Beijing, China; ^2^The Fifth Clinical Medical College of Perking University, Beijing, China

**Keywords:** intertrochanteric fracture, bibliometric, visualized study, co-authorship analysis, co-citation analysis

## Abstract

**Background:** Intertrochanteric femur fractures, which are common geriatric osteoporotic fractures, have imposed a huge economic and social burden. This study clarified the global status of research on intertrochanteric fractures between 2001 and 2020 and predicted future research trends in this field using bibliometric and visualized studies.

**Methods:** Publications related to intertrochanteric fractures were retrieved from the Web of Science (WoS) database. All studies were published between 2001 and 2020. Bibliometric and co-occurrence analyses were conducted using VoS viewer software.

**Results:** In total, 2,632 studies were retrieved. The number of global publications regarding intertrochanteric fractures increased annually. The United States was the largest contributor, ranking first in total publications, citations, and the H-index. Switzerland had the highest average citation frequency among the 10 countries with the highest number of publications. The journal that published the most articles regarding intertrochanteric fractures was the Injury International Journal of The Care of The Injured, with 290 articles published. This journal also ranked first in the citation frequency. MJ Parker, an author, published the most papers in the field, and the University of California research team at San Francisco contributed the most publications in this field. During the co-occurrence analysis, all keywords were divided into four clusters: internal fixation study, complication study, risk-factor study, and survival and prognosis analysis study. The internal fixation and survival and prognosis analysis studies were predicted as the next hot topics in the field of intertrochanteric fractures.

**Conclusions:** Intertrochanteric fractures are gaining increasing research attention according to the current global trend, and the number of publications regarding intertrochanteric hip fractures will continue to increase. The United States currently publishes the most articles on intertrochanteric fractures. The number of studies related to internal fixation, survival, and prognosis analysis is increasing, suggesting that these topics may become the next research hotspots in the area of intertrochanteric fractures.

## Introduction

Intertrochanteric femur fractures, the most common type of fragility fractures in the elderly, account for 55% of proximal femoral fractures (1). As life expectancy and the global elderly population increase, the incidence of hip fractures in the elderly, especially intertrochanteric fractures, will continue to increase (2). Intertrochanteric femur fractures have high fatality and disability rates, resulting in a huge burden on the economy and on society (3–5). Intertrochanteric femur fractures have become an important public health problem.

Intertrochanteric femur fractures are typically treated by orthopedic trauma surgeons, as surgical treatment is often recommended for such patients, especially the elderly. Patients are encouraged to ambulate as soon as possible to reduce the risk of complications and mortality (6). Intertrochanteric fractures can be treated *via* internal fixation with a dynamic hip screw (DHS), percutaneous compression plate (PCCP), proximal femoral locked compression plate, less invasive stabilization system, or intramedullary fixation devices (7–9).

Intertrochanteric femur fractures are now a global public health problem and have imposed a huge economic and social burden. However, the global trends in intertrochanteric femur fracture research are unclear. Publications are important predictors of research trends within a country or institution. Bibliometrics can be used to qualitatively and quantitatively assess research trends based on the bibliometric characteristics of the literature database (10). This method effectively analyzes the development of a specific field and evaluates the related contributions of journals, institutions, and countries. In order to further clear the global research direction of intertrochanteric femur fractures and provide some evidence about the prevention and treatment of these fractures and the formulation of more reasonable medical policies, this study analyzed the current global research status and predicted future research trends for intertrochanteric hip fractures. In this study, bibliometrics were used to clarify the research trends for intertrochanteric femur fractures over the past 20 years and to predict future research hotspots.

## Methods

### Data Sources and Search Strategy

Publications were retrieved from the Web of Science (WoS) and Science Citation Index Expanded (SCIE) databases. The WoS and SCIE databases are the optimal databases for bibliometric data and include over 12,000 high-impact, quality scientific international journals. All the publications included in this study were articles or reviews published in English between 2001 and 2020 and were retrieved from the WoS database (11). The following search terms were used: trochanteric fracture, intertrochanteric fracture, intertrochanteric fractures, intertrochanteric fracture of femur. The search terms of randomized controlled trials (RCTs) were used: trochanteric fracture, intertrochanteric fracture, intertrochanteric fractures, intertrochanteric fracture of femur, and randomized controlled trial NOT meta-analysis.

### Data Screening and Collection

The relevant information of all identified publications, including title, keywords, abstract, year of publication, author, impact factor, H-index, nationality, affiliates, research direction, and funding agencies, was downloaded from the WoS database. Any publication for which the relevant information was not available was excluded from this study. Two authors independently conducted the search and gathered the relevant data. The final screening results were reviewed by a third author.

### Bibliometric Analysis

All identified data were rearranged according to the publication year, country, number of publications, total citations, average citations per term, H-index, journal, author, institution, and research fund direction. The H-index is an author-level metric that measures the productivity and citation impact of publications by an individual author (12, 13). Microsoft Office Excel 2019 (Microsoft Corporation, Santa Rosa, CA, USA) was used to collect and rank all the publication characteristics. The predicted publication growth model equation was derived as a cubic degree polynomial function using an intrinsic function of the Microsoft Office Excel 2019.

### Visualized Study

The bibliometric visualization and analysis of the literature were conducted with the Java program VoS viewer (Leiden University, Leiden, The Netherlands). In this study, the VoS viewer was used to conduct the bibliometric analysis and visualization research co-citation, co-authorship, co-occurrence, and bibliographic coupling. The associations between authors, institutions, and countries were visualized using weighted total link strength (TLS) lines. The TLS represents the total strength of the links of an item with other items, and the higher the TLS, the more weight is used to draw the link on the visual analysis.

The co-citation analysis method is a quantitative intelligence research method used to study the relationships between documents by analyzing the frequency of simultaneous citations of the two documents by other documents. The VoS viewer software is used to calculate the co-citation link strength between two items.

The co-occurrence analysis determines if the relationship between the items is based on the number of publications in which they appear together (14). The purpose of this analysis is to determine research fields and hot issues. It serves as an important indicator for tracking scientific development.

Bibliometric coupling is a more advanced research method of literature coupling. If papers A and B cited the same references, they have a coupling relationship, indicating that their research contents are similar. The VoS viewer was used to analyze the contents of all selected literature.

## Results

### Global Publication Trends

#### Number and Trend of Publications

**Figure 1:**
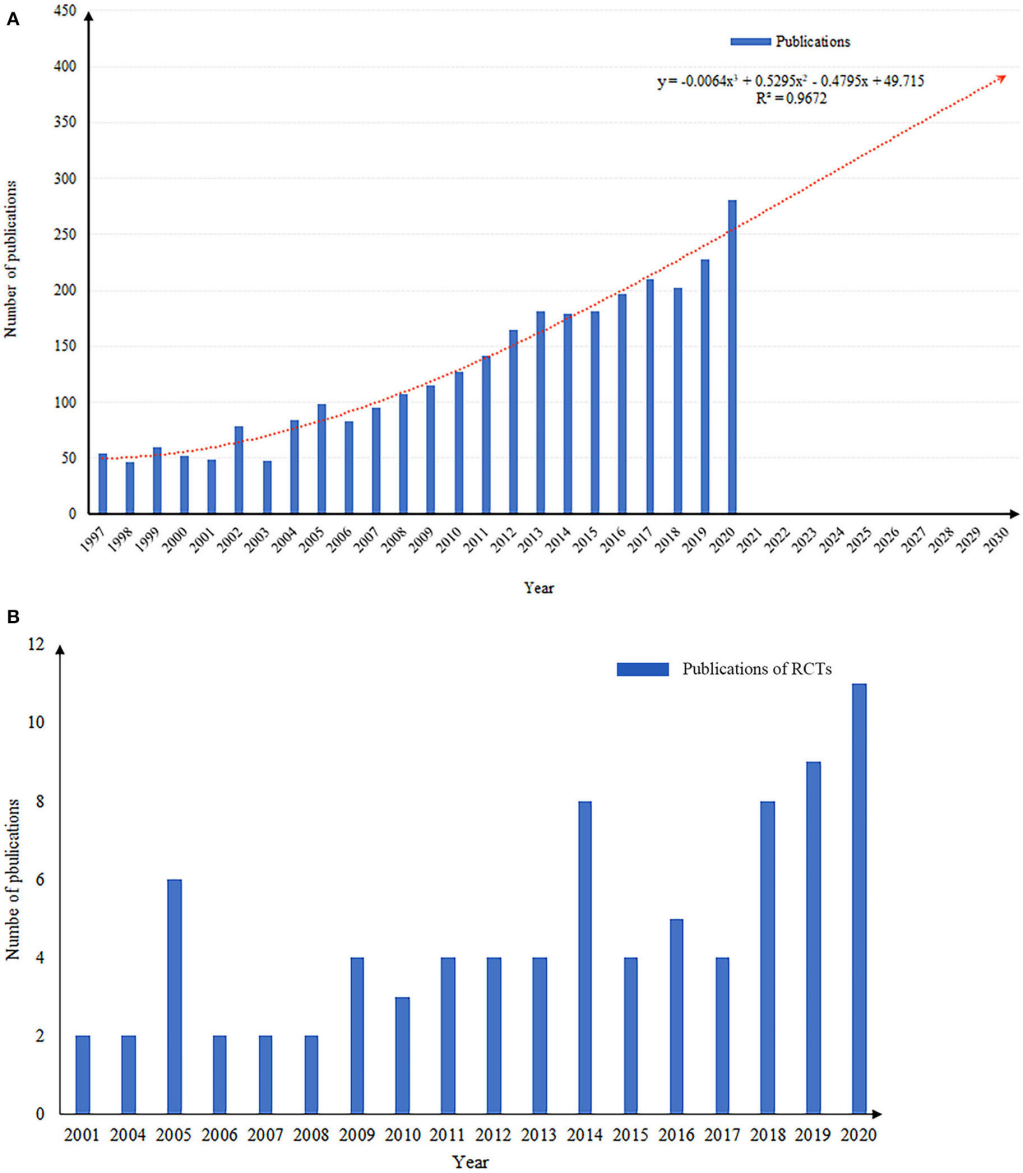
**(A)** The number of publications by year, and the predicted growth curve of intertrochanteric fractures. **(B)** The number of publications of RCTs by year.

The number of publications regarding intertrochanteric fractures increased each year from 1997 to 2020, with the largest number of papers published in 2020 (*n* = 281; 7.6%) (Figure 1A). The predicted growth model equation was *y* = −0.0064*x*^3^ + 0.5295*x*^2^ –.4795*x* + 49.715, *R*^2^ = 0.9672, with *x* representing the year and y representing the predicted number of publications per year. Based on this equation the number of publications in this field is expected to increase to ~400 by 2030. The number of publications of RCTs from 2001 to 2020 is 84 in total, with the most papers published in 2020 (*n* = 11; 13.0%) (Figure 1B).

#### Contribution of Each Country

**Figure 2:**
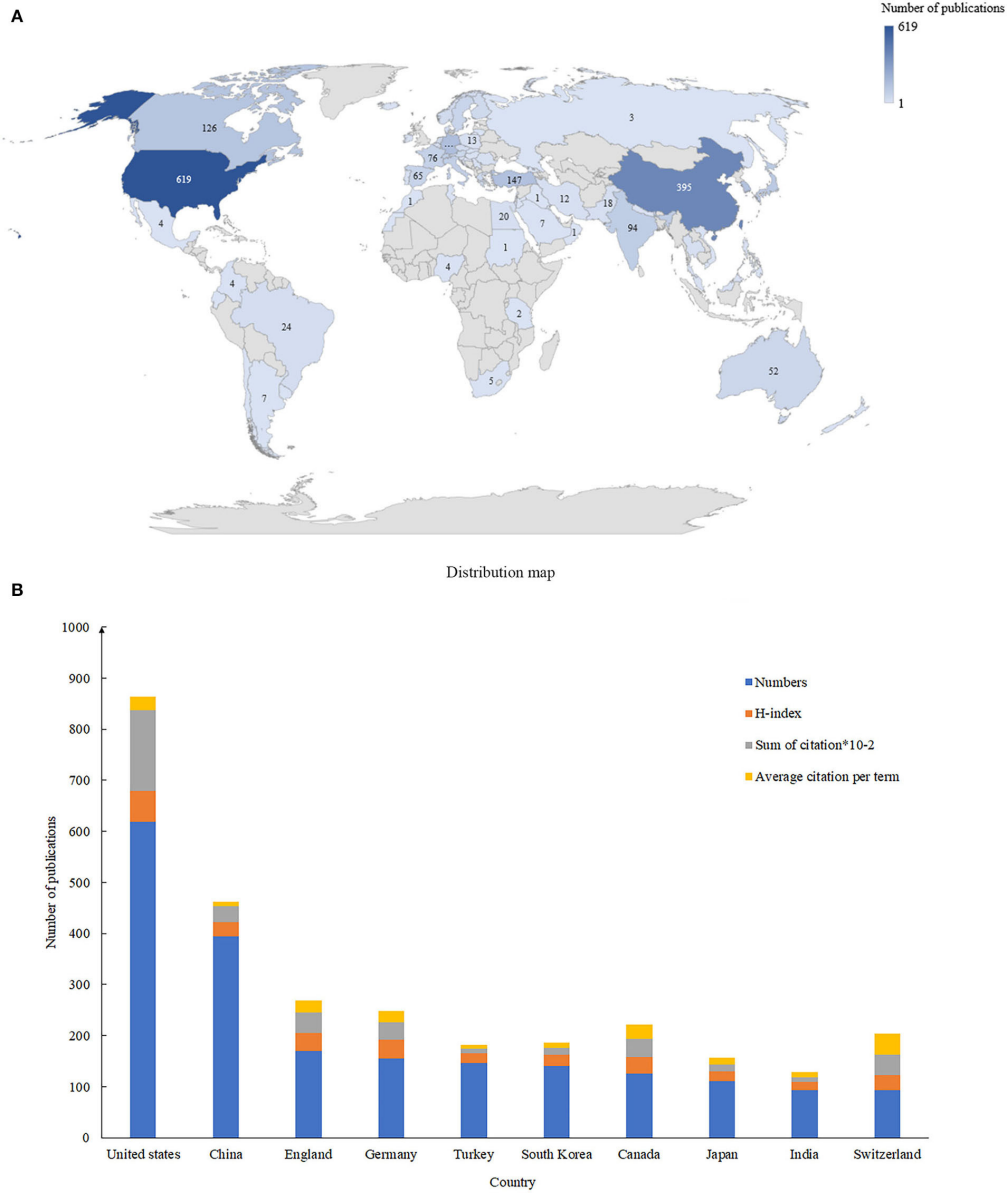
**(A)** The global distribution of countries in intertrochanteric fracture research. **(B)** The top 10 productive and cited countries in intertrochanteric fracture research.

A total of 75 countries contributed published articles regarding intertrochanteric fractures (Figure 2A). The United States had the largest number of publications in this field (*n* = 615; 23.5%) followed by China (*n* = 395; 15.0%) and England (*n* = 170; 6.5%) (Table 1 and Figure 2B).

**Table 1:** The top 10 productive countries in intertrochanteric fracture research.

**Country**	**Number (%)**	**H-index**	**Total citations**	**Average citation per term**
United States	619 (23.5%)	60	15,848	41.46
China	395 (15.0%)	28	3,090	27.66
England	170 (6.5%)	36	4,001	25.6
Germany	156 (5.9%)	36	3,463	23.54
Turkey	147 (5.6%)	18	1,020	22.2
South Korea	141 (5.4%)	21	1,427	11.93
Canada	126 (4.8%)	33	3,485	10.12
Japan	111 (4.2%)	20	1,324	9.65
India	94 (3.6%)	16	907	7.82
Switzerland	94 (3.6%)	29	3,925	6.94

### Quality of the Publications of Each Country

The United States had the highest H-index (H-index = 60) followed by England (H-index = 36), Germany (H-index = 36), and Canada (H-index = 33). The United States also had the most total citations (*n* = 15,848), followed by England (*n* = 4,001), Switzerland (*n* = 3,925), Canada (*n* = 3,485), and Germany (*n* = 3,463). The publications from Switzerland had the highest average number of citations (*n* = 41.46), followed by Canada (*n* = 27.66), England (*n* = 25.6), and Germany (*n* = 23.54).

### Analysis of Global Publication Trends

#### Journals

**Figure 3:**
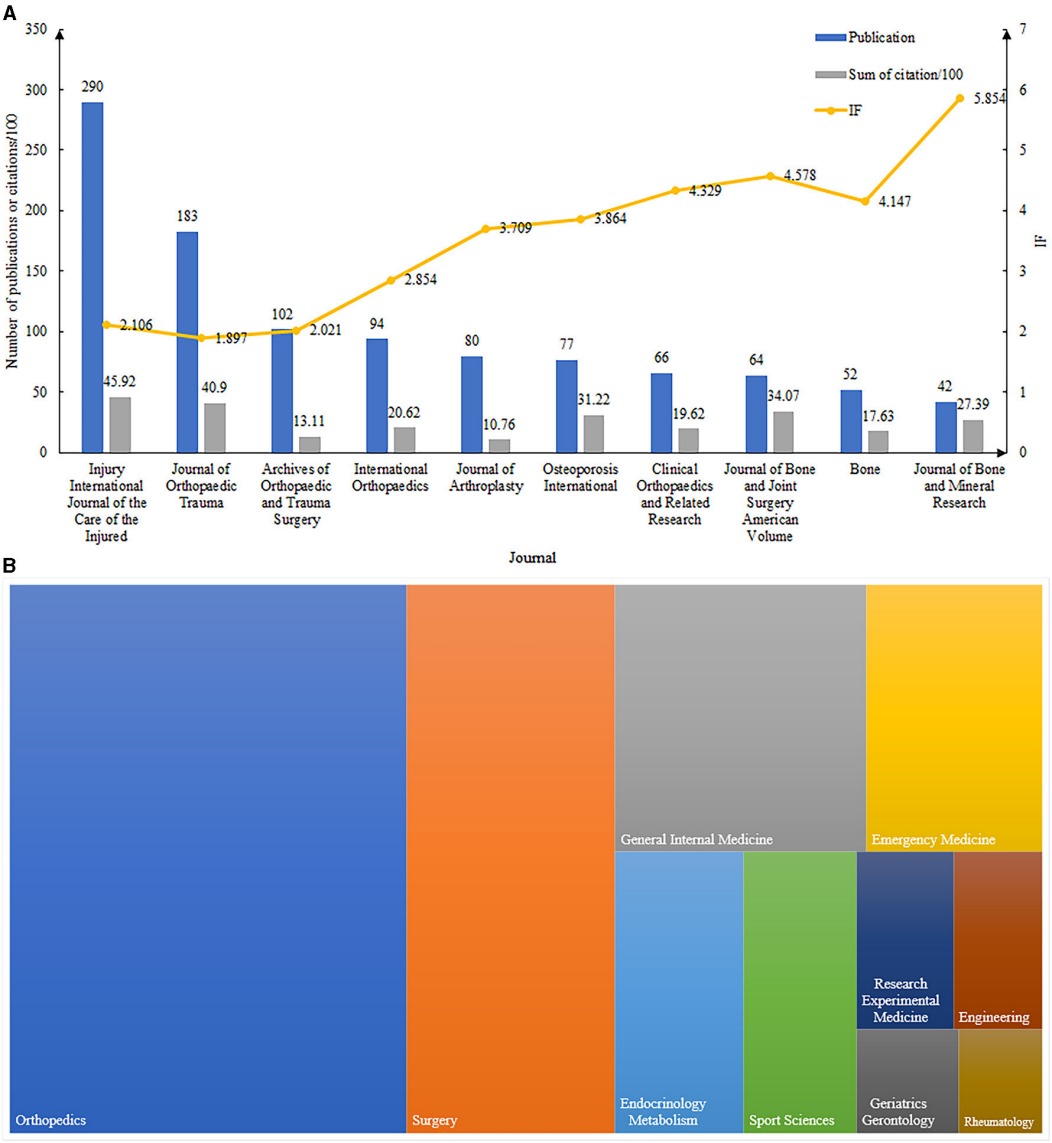
**(A)** The top 10 productive and cited journals in intertrochanteric fracture research. **(B)** The top 10 research directions of intertrochanteric fractures.

Among the journals, the Injury International Journal of the Care of the Injured had the most publications regarding intertrochanteric fractures (*n* = 290) followed by the Journal of Orthopedic Trauma (*n* = 183), Archives of Orthopedic and Trauma Surgery (*n* = 102), and International Orthopedics (*n* = 94) (Figure 3A). Injury International Journal of the Care of the Injured had the highest number of citations (*n* = 4,592) followed by the Journal of Orthopedic Trauma (*n* = 4,090), the Journal of Bone and Joint Surgery, American Volume (*n* = 3,407), and Osteoporosis International (*n* = 3,122). The Journal of Bone and Mineral Research had the highest impact factor (IF; 5.854), followed by the Journal of bone and Joint Surgery, American Volume (IF = 4.578) and Clinical Orthopedics and Related Research (IF = 4.329).

#### Research Directions

The most popular research directions of the publications regarding intertrochanteric fractures were orthopedics (*n* = 1,596; 60.6%), surgery (*n* = 836; 31.8%), general internal medicine (*n* = 493; 18.7%), and emergency medicine (*n* = 344; 13.1%) (Figure 3B).

#### Authors

Martyn J Parker had the most publications regarding intertrochanteric fractures (*n* = 27), followed by Yong-Chan Ha (*n* = 24), Kyung-Hoi Koo (*n* = 20), Timo Jamsa (*n* = 19), and Young-Kyun Lee (*n* = 19) (Table 2).

**Table 2:** The top five productive authors in intertrochanteric fracture research.

**Author**	**Country**	**Number of publications**	**H-index**	**Average citation**	**Total citations**	**Institution**
Parker, Martyn J	England	27 (1.0%)	14	31.26	844	Peterborough City Hospital
Ha, Yong-Chan	South Korea	24 (0.9%)	10	11.5	276	Chung-Ang University, Department of Orthopedic Surgery
Koo, Kyung-Hoi	South Korea	20 (0.7%)	9	12.1	242	Seoul National University (SNU), Department of Orthopedic Surgery
Jamsa, Timo	Finland	19 (0.7%)	7	125	125	University of Oulu Research Unit Medical Imaging Physics & Technology
Lee, Young-Kyun	South Korea	19 (0.7%)	4	113	113	Seoul National University (SNU) Bundang Hospital

#### Institutions

The University of California at San Francisco had the most publications (*n* = 48), followed by the Hospital of Special Surgery in the United States (*n* = 30), and Shanghai Jiao Tong University in China (*n* = 27) (Table 3). The10 institutions with the most publications were mainly located in the United States, China, South Korea, and Canada.

**Table 3:** The top 10 productive institutions in intertrochanteric fracture research.

**Rank**	**Institution**	**Number of publications**	**Country**
1	University of California–San Francisco	48 (1.8%)	USA
2	Hospital of Special Surgery	30 (1.1%)	USA
3	Shanghai Jiao Tong University	27 (1.1%)	China
4	Seoul National University	25 (0.95%)	South Korea
5	Tongji University	25 (0.95%)	China
6	University of Toronto	25 (0.95%)	Canada
7	Fudan University	24 (0.91%)	China
8	Korea University	24 (0.91%)	South Korea
9	Mayo clinic	24 (0.91%)	USA
10	Chung-Ang University	23 (0.87%)	South Korea

#### Funding Organizations

The United States Department of Health and Human Services funded the most publications (*n* = 98; 3.7%), followed by the National Institutes of Health (*n* = 94; 3.6%) and the National Natural Science Foundation of China (*n* = 70; 2.7%) (Table 4). Seven funding institutions in the United States provided funding for publications regarding the intertrochanteric fractures. The remaining funding institutions were located in China, Canada, and Japan.

**Table 4:** The top 10 productive funding agencies in intertrochanteric fracture research.

**Rank**	**Funding agency**	**Number (%)**	**Country**
1	United States of department of health and human services (HHS)	98 (3.7%)	USA
2	National institutes of health (NIH)	94 (3.6%)	USA
3	National Natural Science Foundation of China (NSFC)	70 (2.7%)	China
4	National institute of arthritis and musculoskeletal and skin diseases (NIAMS)	52 (2.0%)	USA
5	National institute on aging (NIA)	47 (1.8%)	USA
6	Natural sciences and engineering research council of Canada (NSERC)	19 (0.7%)	Canada
7	Ministry of education, culture, sports, science and technology (MEXT)	18 (0.7%)	Japan
8	Amgen company	16 (0.6%)	USA
9	National institute for health research (NIHR)	16 (0.6%)	USA
10	Merck company	15 (0.6%)	USA

### Co-authorship Analysis

#### Authors

A total of 150 authors had at least five publications. Yong-Chan Ha had the greatest TLS (TLS = 47), followed by Kyung-Hoi Koo (TLS = 45), Young-Kyun Lee (TLS = 43), Shi-Min Chang (TLS = 27), and Timo Jamsa (TLS = 27).

#### Institutions

A total of 227 institutions had at least five publications. The five institutions with the greatest TLS were the University of California at San Francisco (TLS = 76), University of Pittsburgh (TLS = 40), Oulu University Hospital (TLS = 36), University of Oulu (TLS = 36), and Chung-Ang University (TLS = 34).

#### Countries

A total of 47 countries had at least five publications. The five countries with the greatest TLS were the United States (TLS = 206), England (TLS = 135), Germany (TLS = 133), Switzerland (TLS = 93), and Austria (TLS = 88).

### Bibliometric Coupling Analysis

#### Authors

A total of 150 authors had at least five publications. The five authors with the greatest TLS were Martyn J Parker (TLS = 8,170), Matthias Knobe (TLS = 7,960, Yong-Chan Ha (TLS = 7,265), Jong-Keon Oh (TLS = 7,201), and Kyung-Hoi Koo (TLS = 6,961).

#### Journals

A total of 83 journals had at least five publications. The five journals with the greatest TLS were Injury-International Journal of Care of the Injured (TLS = 148,669), the Journal of Orthopedic Trauma (TLS = 103,297), Archives of Orthopedic and Trauma Surgery (TLS = 49,940), International Orthopedics (TLS = 49,424), and the Journal of Bone and Joint Surgery, American Volume (TLS = 40,827).

#### Institutions

A total of 227 institutions had at least five publications. The five institutions with the greatest TLS were the University of California at San Francisco (TLS = 28,057), University of Oulu (TLS = 15,894), Korea University (TLS = 15,030), Oulu University Hospital (TLS = 14,471), and University of Toronto (TLS = 14,274).

#### Countries

A total of 47 countries had at least five publications. The five countries with the greatest TLS were the United States (TLS = 269,857), China (TLS = 195,221), Germany (TLS = 124,862), England (TLS = 103,990), and Turkey (TLS = 87,719).

### Co-citation Analysis

#### Journals

**Figure 4:**
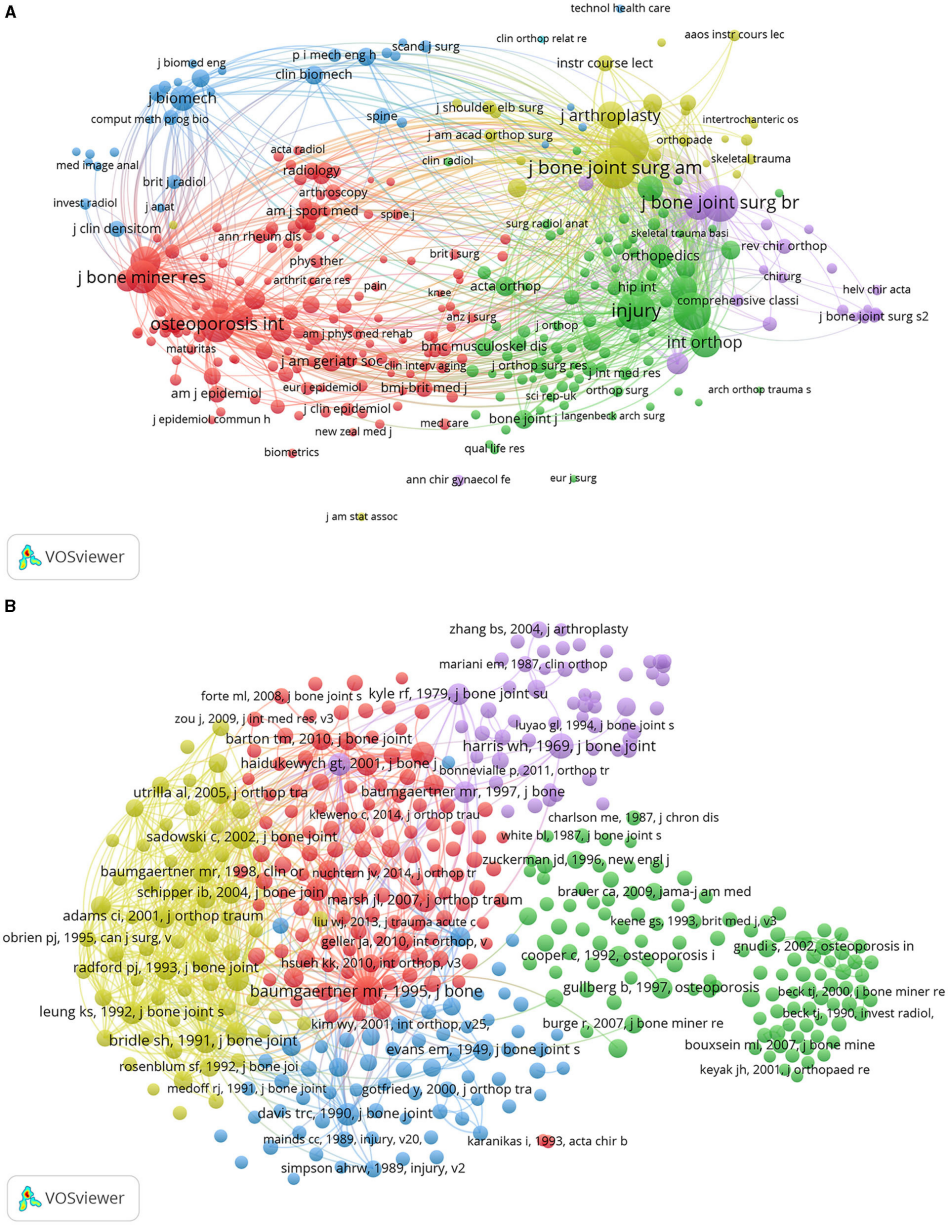
Co-citation analysis of global research on intertrochanteric fractures. **(A)** Co-citation analysis map of journals on intertrochanteric fractures. **(B)** Co-citation analysis map of publications on intertrochanteric fractures.

There were 320 journals that were cited at least 20 times. The five journals with the greatest TLS were the Journal of Bone and Joint Surgery, American Volume (TLS = 168,229), Clinical Orthopedics and Related Research (TLS = 143,503), Injury International Journal of Care of the Injured (TLS = 140,542), the Journal of Orthopedic Trauma (TLS = 130,033), and the Journal of Bone and Joint Surgery, British Volume (TLS = 115,941) (Figure 4A).

#### Publications

A total of 429 publications were cited at least 20 times. The four publications with the greatest TLS were Cool (15) (TLS = 4,447), Bridle et al. (16) (TLS = 3,087), Hardy et al. (17) (TLS = 2,775), and Adams et al. (18) (TLS = 2,669 times) (Figure 4B).

### Co-occurrence Analysis

**Figure 5:**
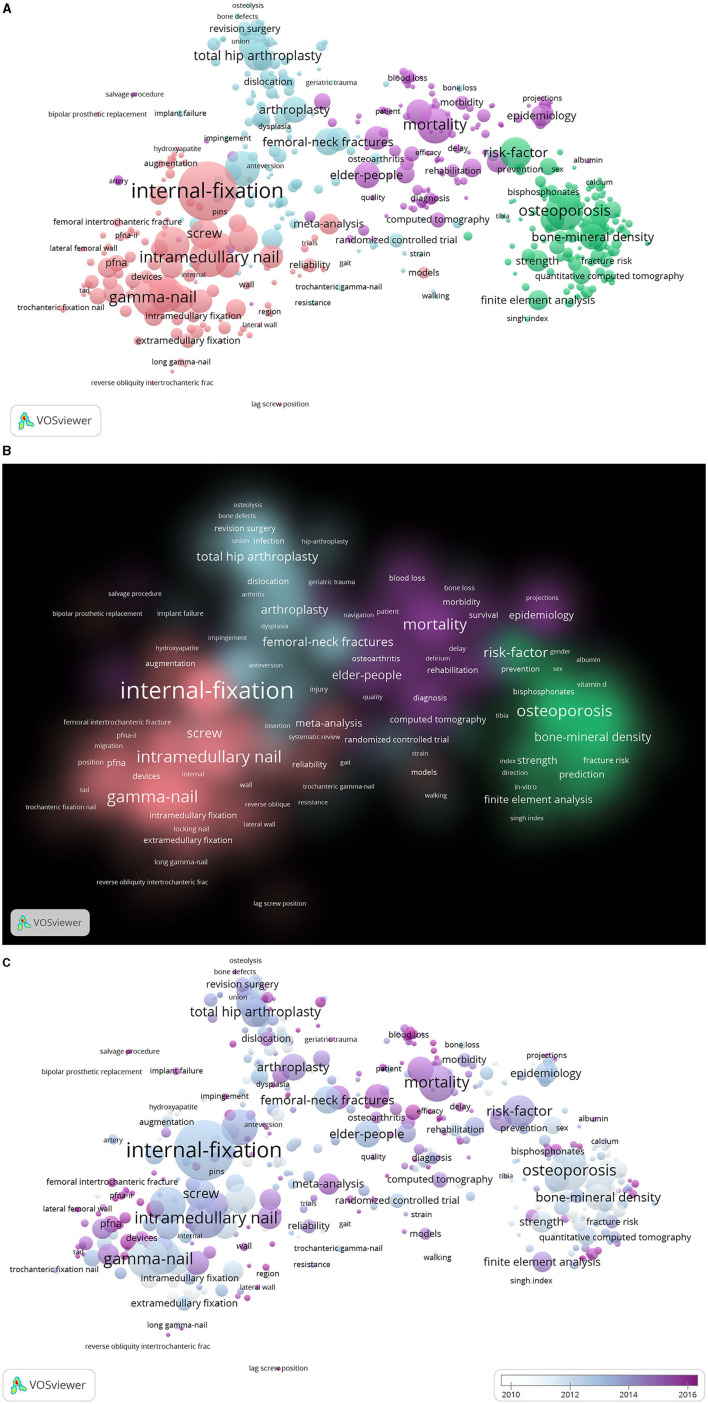
Co-occurrence analysis of global research on intertrochanteric fractures. **(A)** Mapping of keywords in the research. **(B)** Density distribution of keywords clusters. **(C)** Average year map of keywords.

A total of 744 keywords were used at least five times (Figure 5A). The keywords were divided into four clusters: internal-fixation study (pink), complication study (blue), risk-factor study (green), and survival and prognosis analysis study (purple) (Figure 5B). In the internal-fixation study cluster, the most frequently used keywords were intramedullary nail, gamma nail, dynamic hip screw, and failure. In the complication study cluster, the most frequently used keywords were replacement, arthroplasty, follow up, and management. In the risk-factor study cluster, the most frequently used keywords were osteoporosis, bone mineral density, postmenopausal women, and finite element analysis. In the cluster of survival and prognosis analysis study cluster, the most frequently used keywords were mortality, outcomes, elder-people, and epidemiology.

The keywords were then categorized based on when they appeared in the literature (Figure 5C). Before 2010, most studies were risk-factor studies or complication studies. From 2010 to 2020, internal-fixation studies and survival and prognosis analysis studies became more prominent in this field.

## Discussion

### Global Trends in Intertrochanteric Fracture Research

Bibliometric and visualized studies show the current status and predict the future development trends in research fields. This study presents a comprehensive overview of the trends and development of scientific output for intertrochanteric femur fractures from 2001 to 2020. The number of articles related to intertrochanteric fractures has increased nearly 6-fold since 2001. It is estimated that the number of publications in this field will increase to ~400 by 2030, indicating that this field will continue to be a research hotspot. The increasing number of publications is related to the increasing economic and social burden of intertrochanteric fractures, which also indicates that the research in this field will be thorough and comprehensive.

### Status and Quality of Global Publications

The H-index and the total number of citations are important indicators of the academic impact and quality of a publication (12). The United States has the most publications and citations and the highest average citation frequency, indicating that this country contributes most to this field. China has also made a significant contribution in this field. Some countries such as England, Germany, Canada, and Switzerland have also played an important role based on their high H-indices and average citation frequencies. However, most countries in Southeast Asia and Africa do not contribute to this field, and their lower economic level may help explain that. Conversely, the reason for the high volume of research from a small country like Switzerland is that their developed economy can support extensive scientific research, and their lower population mobility.

Four journals (*Injury International Journal of the Care of the Injured*, the *Journal of Orthopedic Trauma, Archives of Orthopedic and Trauma Surgery*, and *International Orthopedics*) were identified as having published the most studies on intertrochanteric fractures. The total number of publications by these four journals accounted for approximately one-fourth of all publications in this field, indicating that future findings in this field are likely to be reported in these journals. Among the 10 journals with the most publications, an increase in the total number of publications tended to reduce the journal's impact factor in the short term.

Nearly all of the 10 institutions with the most publications were located in the United States, China, South Korea, and Canada, and seven of the 10 organizations that funded the most number of studies were located in the United States. These results indicate that first-class research institutions and adequate financial support are essential for improving a country's overall academic contributions.

Martyn J Parker, Yong-Chan Ha, Kyung-Hoi Koo, Timo Jamsa, and Young-Kyun Lee published the most articles in this field. Their publications have had a major impact on intertrochanteric fractures. Their future research will continue to have a significant impact on the development of this field. Their research will likely reflect the latest developments in the field of intertrochanteric fractures.

The bibliographic analysis identified the United States and *Injury International Journal of the Care of the Injured* as the country and journal that published the most related articles. The *Journal of Bone and Joint Surgery, American Volume* was the most cited journal in the field of intertrochanteric fracture research, indicating that this is a landmark journal that is likely to publish future research in this field.

However, the increasing literature has not led to any changes in outcomes. In addition, the number of high-quality RCT studies were quite low (85/2632; 3.23%) in the past 20 years, and this was also the case in 2020 (11/281; 3.91%). The possible reasons are as follows: (1) Because of the limitation associated with some medical conditions and, many underdeveloped countries lack the registration systems suitable for patients with hip fractures. (2) Furthermore, most published publications are low-quality retrospective studies, and high-quality RCTs and other functional publications are relatively lacking. (3) In addition, the community is guilty of performing a lot of research on topics that do not really matter in the overarching scheme of the patient's quality of life.

### Research Focus on Intertrochanteric Fractures

The research directions and hotspots in the field of intertrochanteric fractures were identified using the co-occurrence analysis. The keywords were categorized based on study type, and the most frequently used keywords were associated with internal fixation and survival and prognostic analysis studies. Therefore, these study types are the hotspots in current research. However, this result may also indicate that these topics require additional research.

The overlay visualization map was color-coded by the VOS viewer based on the average year that the keywords appeared in publications (19), which is a method used to monitor the progress of research. The results of this analysis indicate that internal fixation studies and survival and prognosis analysis studies may be the next hotspots for intertrochanteric fracture research. In recent years, DHS, gamma nails, proximal femoral nails, and inter-Tan have become research hotspots (1, 7, 20, 21). In addition, mechanical failure of internal fixation, such as rotation, incision, and migration of internal fixation, has also attracted the attention of researchers (22–26). Additional research is required to overcome these complications and further improve the internal fixation devices for intertrochanteric fractures. In addition, an increasing number of studies have focused on issues such as perioperative risk assessment, comprehensive management of perioperative patients, and how to reduce the mortality and disability rates of patients with intertrochanteric fractures (27).

The results of this study indicate that the number of publications regarding intertrochanteric fractures has increased in the past two decades and that this trend will continue in the future. Intertrochanteric fractures will continue to be a hot topic in the field of orthopedics. The bibliometric and visualized analyses provide investigators with data regarding the leading countries, authors, institutions, and publications in this field, which will help researchers quickly understand the most advanced research and discoveries in this field. In addition, the co-occurrence analysis and overlay visualization map present the research hotspots and future research directions, which may help funding agencies and public health policy makers make more decisions regarding intertrochanteric fractures.

### Limitations

The bibliometric analysis included in this study is limited by the use of one database. The scopes of the major databases differ, which may lead to some publications being omitted from the analysis when only one database is used. Additionally, only English-language studies were included, which may lead to a language bias. The impact of more recent publications may have been underestimated due to the low number of citations. Therefore, researchers must be aware of the latest published literature and literature published in languages other than English.

## Conclusions

This study clarified the global research status for intertrochanteric fractures between 2001 and 2020 and predicted future research trends in this field. The United States contributed the most publications and citations, whereas the Injury International Journal of the Care of the Injured can be considered a landmark journal as it has published the most papers related to this field. Studies regarding the survival, prognosis, and mechanical failure of internal fixations will be the research hotspots in the future. In future research, we need to carry out more high-quality RCTs research. In addition, we call on countries such as Southeast Asia and Africa to establish standardized hip fracture registration systems as soon as possible, which will help further improve the global hip fracture prevention and treatment system.

## Data Availability Statement

The original contributions presented in the study are included in the article/supplementary material, further inquiries can be directed to the corresponding author.

## Author Contributions

LW: project administration, validation data curation, writing—original draft preparation, supervision, and funding acquisition. ZZ: conceptualization, methodology, data curation, formal analysis, and writing—original draft. YQ: methodology, writing—review, and editing. YWZ: data curation and writing—original draft. YZ and FS: visualization and validation. JL: data curation, writing—review, and editing. TZ: data curation and project administration. All authors contributed to the article and approved the submitted version.

## Funding

This study was supported by Non-profit Central Research Institute Fund of Chinese Academy of Medical Sciences (2021-JKCS-021).

## Conflict of Interest

The authors declare that the research was conducted in the absence of any commercial or financial relationships that could be construed as a potential conflict of interest.

## Publisher's Note

All claims expressed in this article are solely those of the authors and do not necessarily represent those of their affiliated organizations, or those of the publisher, the editors and the reviewers. Any product that may be evaluated in this article, or claim that may be made by its manufacturer, is not guaranteed or endorsed by the publisher.
