# Corrigendum: Global Trends in Intertrochanteric Hip Fracture Research From 2001 to 2020: A Bibliometric and Visualized Study

**DOI:** 10.3389/fsurg.2022.865369

**Published:** 2022-02-18

**Authors:** Ze Zhang, Yudian Qiu, Yawen Zhang, Yi Zhu, Fengpo Sun, Junchuan Liu, Tongyi Zhang, Liangyuan Wen

**Affiliations:** ^1^Department of Orthopedics, Beijing Hospital, National Center of Gerontology, Institute of Geriatric Medicine, Chinese Academy of Medical Sciences, Beijing, China; ^2^The Fifth Clinical Medical College of Perking University, Beijing, China

**Keywords:** intertrochanteric fracture, bibliometric, visualized study, co-authorship analysis, co-citation analysis

There is an error in the Funding statement. The correct number for **JC2021CL011** is **2021-JKCS-021**.

There is an error in the Funding statement. The correct Name for the Funder is **Non-profit Central Research Institute Fund of Chinese Academy of Medical Sciences**.

The authors apologize for this error and state that this does not change the scientific conclusions of the article in any way. The original article has been updated.

## Publisher's Note

All claims expressed in this article are solely those of the authors and do not necessarily represent those of their affiliated organizations, or those of the publisher, the editors and the reviewers. Any product that may be evaluated in this article, or claim that may be made by its manufacturer, is not guaranteed or endorsed by the publisher.

